# A comparative analysis of national HIV policies in six African countries with generalized epidemics

**DOI:** 10.2471/BLT.14.147215

**Published:** 2015-04-28

**Authors:** Kathryn Church, Francis Kiweewa, Aisha Dasgupta, Mary Mwangome, Edith Mpandaguta, Francesc Xavier Gómez-Olivé, Samuel Oti, Jim Todd, Alison Wringe, Eveline Geubbels, Amelia Crampin, Jessica Nakiyingi-Miiro, Chika Hayashi, Muthoni Njage, Ryan G Wagner, Alex Riolexus Ario, Simon D Makombe, Owen Mugurungi, Basia Zaba

**Affiliations:** a Department of Population Health, London School of Hygiene & Tropical Medicine, Keppel St, London, WC1E 7HT, England.; b Makerere University School of Public Health, Kampala, Uganda.; c Ifakara Health Institute, Ifakara, United Republic of Tanzania.; d Manicaland HIV/STD Prevention Project, Harare, Zimbabwe.; e Agincourt, Rural Public Health and Health Transitions Research Unit, Faculty of Health Sciences, University of the Witwatersrand, Johannesburg, South Africa.; f African Population Health Research Center, Nairobi, Kenya.; g London School of Hygiene & Tropical Medicine, London, England.; h MRC/UVRI Uganda Research Unit on HIV/AIDS, Entebbe, Uganda.; i World Health Organization, Geneva, Switzerland.; j Consultant, Nairobi, Kenya.; k Ministry of Health, Kampala, Uganda.; l Ministry of Health, Lilongwe, Malawi.; m Ministry of Health and Child Care, Harare, Zimbabwe.

## Abstract

**Objective:**

To compare national human immunodeficiency virus (HIV) policies influencing access to HIV testing and treatment services in six sub-Saharan African countries.

**Methods:**

We reviewed HIV policies as part of a multi-country study on adult mortality in sub-Saharan Africa. A policy extraction tool was developed and used to review national HIV policy documents and guidelines published in Kenya, Malawi, South Africa, Uganda, the United Republic of Tanzania and Zimbabwe between 2003 and 2013. Key informant interviews helped to fill gaps in findings. National policies were categorized according to whether they explicitly or implicitly adhered to 54 policy indicators, identified through literature and expert reviews. We also compared the national policies with World Health Organization (WHO) guidance.

**Findings:**

There was wide variation in policies between countries; each country was progressive in some areas and not in others. Malawi was particularly advanced in promoting rapid initiation of antiretroviral therapy. However, no country had a consistently enabling policy context expected to increase access to care and prevent attrition. Countries went beyond WHO guidance in certain areas and key informants reported that practice often surpassed policy.

**Conclusion:**

Evaluating the impact of policy differences on access to care and health outcomes among people living with HIV is challenging. Certain policies will exert more influence than others and official policies are not always implemented. Future research should assess the extent of policy implementation and link these findings with HIV outcomes.

## Résumé

**Objectif:**

Comparer les politiques nationales de lutte contre le virus de l'immunodéficience humaine (VIH) qui influencent l'accès aux services de dépistage et de traitement du VIH dans six pays d'Afrique subsaharienne.

**Méthodes:**

Nous avons examiné les politiques de lutte contre le VIH dans le cadre d'une étude multi-pays sur la mortalité des adultes en Afrique subsaharienne. Un outil d'extraction de données a été mis au point et utilisé afin d'examiner les documents et les directives des politiques nationales de lutte contre le VIH publiés en Afrique du Sud, au Kenya, au Malawi, en Ouganda, en République-Unie de Tanzanie et au Zimbabwe entre 2003 et 2013. Des entretiens avec des informateurs clés ont permis de combler les carences de ces résultats. Les politiques nationales ont été classées suivant leur degré de correspondance, explicite ou implicite, avec 54 indicateurs relatifs aux politiques, déterminés d'après des analyses documentaires et des examens d'experts. Nous avons également comparé les politiques nationales avec les recommandations de l'Organisation mondiale de la Santé (OMS).

**Résultats:**

Nous avons observé de grandes différences entre les politiques de ces pays ; chaque pays était avancé dans certains domaines et pas dans d'autres. Le Malawi l'était particulièrement en matière de promotion du démarrage rapide du traitement antirétroviral. Cependant, aucun pays n'avait un contexte politique pouvant systématiquement permettre d'augmenter l'accès aux soins et d'éviter l'arrêt du traitement. Dans certains domaines, les pays allaient plus loin que les recommandations de l'OMS et les informateurs ont indiqué que la pratique dépassait souvent le cadre des politiques.

**Conclusion:**

Évaluer l'impact des différentes politiques sur l'accès aux soins et les résultats en termes de santé des personnes qui vivent avec le VIH n'est pas chose simple. Certaines politiques exercent une influence plus forte que d'autres et les politiques officielles ne sont pas toujours mises en œuvre. Des recherches ultérieures devraient évaluer le degré de mise en œuvre des politiques et mettre en lien leurs conclusions avec les résultats de la lutte contre le VIH.

## Resumen

**Objetivo:**

Comparar las políticas nacionales relativas al virus de la inmunodeficiencia humana (VIH) que influencian el acceso a las pruebas del VIH y a los tratamientos en seis países sub-saharianos.

**Métodos:**

Se revisaron las políticas relativas al VIH como parte de un estudio multinacional sobre la mortalidad de adultos en África Subsahariana. Se desarrolló una herramienta de extracción de políticas y se utilizó para revisar los documentos y guías de las políticas nacionales relativas al VIH publicadas en Kenia, Malawi, República Unida de Tanzania, Sudáfrica, Uganda y Zimbabue entre 2003 y 2013. Se hicieron entrevistas a informantes claves que ayudaron a llenar los vacíos en los resultados. Las políticas nacionales se clasificaron según si se adhirieron explícita o implícitamente a 54 indicadores de políticas, identificados mediante bibliografía y opiniones de expertos. Asimismo, se compararon las políticas nacionales con las directrices de la Organización Mundial de la Salud (OMS).

**Resultados:**

Se descubrió que había una amplia variedad entre las políticas de los países. Cada país estaba más avanzado en algunas áreas que en otras. Malawi estaba especialmente avanzado en la promoción de empezar rápidamente la terapia antirretroviral. Sin embargo, ningún país tenía un contexto de introducción de políticas consistente que incrementara el acceso a la atención primaria y evitara la deserción. Algunos países iban más allá de las orientaciones de la OMS en algunas áreas e informantes clave informaron de que la práctica a menudo superaba la política.

**Conclusión:**

Evaluar el impacto de las diferencias en las políticas relativas en el acceso a la atención primaria y los resultados en la salud entre aquellas personas con VIH es un reto. Algunas políticas ejercerán más influencia que otras y las políticas oficiales no siempre se aplican. Las investigaciones futuras deberían evaluar el grado de aplicación de las políticas y vincular estos resultados con los resultados del VIH.

## ملخص

الغرض **: ** مقارنة السياسات الوطنية لمكافحة فيروس العوز المناعي البشري(HIV) والتي تؤثر على القدرة على الاستفادة من خدمات الاختبار والعلاج من فيروس العوز المناعي البشري في ستة بلدان أفريقية واقعة جنوبي الصحراء.

الطريقة **: ** لقد راجعنا سياسات مكافحة فيروس العوز المناعي البشري كجزء من دراسة شملت العديد من البلدان حول معدلات وفيات البالغين في البلدان الأفريقية الواقعة جنوبي الصحراء. تم إعداد واستخدام أداة استخلاص السياسة لمراجعة الوثائق والمبادئ التوجيهية لسياسة مكافحة فيروس العوز المناعي البشري الوطنية التي نشرت في كينيا وملاوي وجنوب أفريقيا وأوغندا وجمهورية تنزانيا المتحدة وزمبابوي في الفترة ما بين عام 2003 و2013. ولقد ساعدت مقابلات المبلّغين الرئيسين على سد الثغرات في النتائج. صُنِّفت السياسات الوطنية وفقًا لما إذا كانت التزمت صراحةً أو ضمنيًا بـ 54 من مؤشرات السياسة، التي تم تحديدها من خلال المؤلفات الطبية ومراجعات الخبراء. وقمنا أيضًا بمقارنة السياسات الوطنية مع توجيهات منظمة الصحة العالمية (WHO).

النتائج **: ** كان هناك تفاوت كبير في السياسات بين البلدان؛ حيث تميز كل بلد بالتقدم في بعض الجوانب دون غيرها. كانت ملاوي متقدمة على وجه الخصوص في تعزيز فكرة الإسراع في بدء العلاج باستخدام مضادات الفيروسات القهقرية. ومع ذلك، لم يكن لدى أي بلد سياق لتطبيق سياسة تتيح تطوير القدرات بصورة ثابتة، بحيث يُنتظر منه تيسير سبل الاستفادة من الرعاية والحد من الاستنزاف. قامت البلدان بتجاوز توجيهات منظمة الصحة العالمية في مناطق معينة وذكر المبلّغون الرئيسون أن الممارسة غالبًا ما كانت تتجاوز السياسة.

الاستنتاج **: ** من العسير تقييم تأثير اختلافات السياسة في فرص الحصول على الرعاية والنتائج الصحية بين الأشخاص المصابين بفيروس العوز المناعي البشري. فسوف تحقق بعض السياسات المعينة المزيد من التأثير دون سواها، كما لا يتم دائمًا تنفيذ السياسات الرسمية. ينبغي على الأبحاث المستقبلية تقييم مدى تنفيذ السياسات وربط هذه النتائج بحصائل الإصابة بفيروس العوز المناعي البشري.

## 摘要

**目的:**

旨在对比在撒哈拉沙漠以南的六个非洲国家中能够影响艾滋病病毒测试和治疗服务的国家艾滋病病毒 (HIV) 政策。

**方法:**

在撒哈拉沙漠以南的非洲国家中进行有关成人死亡率的多国研究期间,我们评审了艾滋病病毒政策。 当时开发了一种政策提取工具并将其用于评审津巴布韦、肯尼亚、马拉维、南非、坦桑尼亚联合共和国以及乌干达在 2003 年和 2013 年期间出版的国家艾滋病病毒政策文件和指南。关键知情人访谈帮助我们弥补了调查结果中的不足之处。 我们依据国家政策是否以明确或隐含的方式遵循 54 项政策指标来对其进行分类,并且通过文献和专家评审加以确认。 同时,我们还对比了国家政策和世界卫生组织 (WHO) 指南。

**结果:**

各国的政策差异很大;每个国家都在一些方面达到先进水平,而在另一些方面却没有达到先进水平。 马拉维在推动抗逆转录病毒疗法的快速启动方面尤为先进。 然而,没有一个国家能够终保持一贯有利的政策环境,难以按预期提高护理普及率和预防消耗。 各国在某些方面超出了世界卫生组织指南的范围,并且据关键知情人报告,实际情况往往超越政策范围。

**结论:**

评估政策差异对艾滋病病毒患者的护理普及率和医疗效果的影响具有挑战性。 某些政策会比其他政策产生更大的影响,并且官方政策并不总是能够得到落实。 今后的研究应评估政策实施的程度,并将这些调查结果与艾滋病病毒的医疗效果联系起来。

## Резюме

**Цель:**

Сравнить политику в отношении вируса иммунодефицита человека (ВИЧ) в шести странах Африки к югу от Сахары. Оценивается доступность анализа на ВИЧ и услуг по лечению.

**Методы:**

Политики в отношении ВИЧ были рассмотрены в рамках многонационального исследования смертности взрослого населения в странах Африки к югу от Сахары. Для анализа политик был разработан специальный инструмент, который использовался для проверки официальных документов и рекомендаций, связанных с государственной политикой в отношении ВИЧ, опубликованных в Кении, Малави, Южной Африке, Уганде, Объединенной Республике Танзания и Зимбабве в период между 2003 и 2013 гг. Неясные моменты уточнялись благодаря ключевым информаторам. Национальные политики распределялись по категориям в зависимости от того, придерживались ли они явным или косвенным образом 54 показателей, выявленных в ходе оценки публикаций и отчетов специалистов. Было также проведено сравнение национальных политик с рекомендациями Всемирной организации здравоохранения (ВОЗ).

**Результаты:**

Политики в разных странах отличались друг от друга; в каждой из стран отмечался прогресс в одних областях и отставание в других. В частности, Малави значительно выделялась продвижением раннего начала антиретровирусной терапии. Тем не менее ни в одной стране не была реализована эффективная политика, которая облегчала бы доступ к медицинской помощи и препятствовала бы оттоку персонала. В некоторых областях страны пошли дальше рекомендаций ВОЗ и основные информаторы сообщили, что практика часто опережала политику.

**Вывод:**

Оценить, насколько отличия в государственной политике влияют на доступ к лечению и результаты мероприятий по охране здоровья для лиц, живущих с ВИЧ, довольно сложно. Некоторые из политик имеют сравнительно больший эффект, и официальная политика не всегда выполняется. В будущих исследованиях необходимо оценить степень выполнения политик и связать полученные данные с результатами лечения ВИЧ.

## Introduction

By the end of 2012, more than 7.5 million of the estimated 23.5 million people living with human immunodeficiency virus (HIV) in Africa were receiving treatment, compared to only 50 000 people a decade before.^1^ The scale-up of treatment services in such a short period of time has been remarkable. Recent evidence suggests that HIV-attributable mortality has declined by more than 50% since antiretroviral therapy (ART) became available.^2^^–^^8^

Nonetheless, considerable concerns remain regarding high attrition rates throughout the continuum of care from HIV diagnosis, pre-ART care, timely initiation of ART and long-term retention in treatment.^9^ Various studies have observed substantial drop-out of people living with HIV across this care cascade. A recent pooled analysis of 37 studies in sub-Saharan Africa indicates that among those knowing their status, only 57% completed ART eligibility assessment, 66% of those eligible initiated ART and 65% of those initiating treatment were retained on ART.^10^

The network for analysing longitudinal population-based data on HIV in Africa (ALPHA) is investigating the extent of declines in HIV-related adult mortality attributable to treatment and the distribution of deaths at each stage of the diagnosis-to-treatment cascade.^11^^–^^14^ The network collects community-based data from 10 health and demographic surveillance sites in six countries with generalized epidemics – Kenya, Malawi, South Africa, Uganda, the United Republic of Tanzania and Zimbabwe. Table 1 identifies the ALPHA network sites and provides contextual information on the epidemic and treatment programme in each country.

**Table 1:** Characteristics of the HIV epidemic in the six African countries^9^^, ^^15^^-^^24^

Characteristic	Kenya	Malawi	South Africa	Uganda	United Republic of Tanzania	Zimbabwe
Demographic surveillance site(s)	Nairobi and Kisumu	Karonga	Agincourt and UmKhanyakude	Masaka and Rakai	Ifakara and Kisesa	Manicaland
Adult HIV prevalence in 2013^15^	6.0	10.3	19.1	7.4	5.0	15.0
Year of public sector ART introduction	2006	2004	2003/2004	2004	2003/2004	2004
Adult ART coverage in 2013^a^ % (range)^15^	42 (39–46)	51 (48–53)	42 (40–43)	40 (38–43)	41 (38–44)	51 (49–53)
PMTCT coverage in 2013^b^ % (range)^15^	63 (55–72)	79 (71–88)	90 (83– 95)	75 (68–85)	73 (65–83)	78 (70–87)
Adults knowing their HIV status^c^ %	36–56^16^^,^^17^	28^18^	36–55^19^	56^20^	54.5^21^	46.5^22^
Pregnant women knowing their HIV status in 2013^23^	88	76	93	> 95	70	> 95
Donor funding as a proportion of total HIV/AIDS budget in 2013^9^	75–100	75–100	0–24	75–100	50–74	75–100
Doctors per 100 000 people^24^	1.8	0.2	7.6	0.1	1.2	0.6

AIDS: acquired immunodeficiency syndrome; ART: antiretroviral therapy; HIV: human immunodeficiency virus; PMTCT: prevention of mother-to-child transmission.

^a^ Defined as the percentage of adults (15 years and older) living with HIV and receiving ART.

^b^ Defined as the estimated percentage of pregnant women living with HIV who received antiretroviral medicines for PMTCT.

^c^ Defined as testing at least once in the last 12 months and receiving results. Note that dates of studies vary.

To interpret site- and country-specific differences in mortality rates across the diagnosis-to-treatment cascade, we analysed national HIV policies. It is helpful for the agencies that define programme priorities and analyse differences in outcomes to understand the incentives and barriers to accessing – and remaining on – ART in different contexts. Our analysis focuses on the policy response to the HIV epidemic. We have not investigated the sociocultural barriers within communities that influence access to services in different sites.

## Methods

**Fig. 1:**
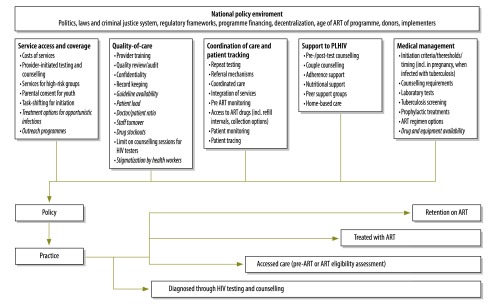
Conceptual framework of HIV policy and service factors influencing HIV-related adult mortality

ART: antiretroviral therapy; HIV: human immunodeficiency virus. PLHIV: people living with HIV.

Note: Factors in italics are only measurable and comparable through facility-based research and are not included in this review.

A conceptual framework was developed, identifying key HIV policy and programmatic factors that may influence HIV-related adult mortality (Fig. 1). These factors were derived from a review of the literature (including a recent systematic review on health sector interventions to ensure a continuum of care),^10^ an initial review of World Health Organization (WHO) guidelines and expert review of indicators by 28 HIV researchers and clinicians. Through the literature review and preliminary analysis of ALPHA network mortality data, we identified three attrition points to inform the structure of our policy review: (i) access to HIV testing and counselling; (ii) access to HIV care and treatment (including assessment of eligibility for treatment initiation and initiation itself); and (iii) retention on ART. Across these three attrition points (diagnosis, HIV care and retention), relevant factors fell into the following five areas: (i) service access and coverage; (ii) quality of care; (iii) coordination of care and patient tracking; (iv) medical management; and (v) support to people living with HIV.

A policy extraction tool was developed to facilitate the indicator review (available from the author). Documents were searched online through ministry of health and national HIV organization websites and/or retrieved in person from official offices and libraries, using the following inclusion criteria: (i) nationally relevant (not clinic- or district-specific); (ii) containing programmatic or clinical guidance on one of the three key adult HIV services: HIV testing and counselling, prevention of mother-to-child transmission (PMTCT) or HIV care and treatment; (iii) published between January 2003 and June 2013. Documents likely to fit these three criteria were considered, including policy statements, clinical guidelines, training manuals, strategies, indicator guides and parliamentary acts. Guideline documents produced by WHO relevant to criteria (ii) and (iii) were also included.

The policy extraction tool was used to collect information on policy content, source, year and policy changes over time. Country teams completed gaps in data collection using unstructured informal interviews with key informants. Informants were either regional or national policy-makers, researchers or clinicians involved in policy development.

We summarized key policies judged most likely to affect access to HIV testing, access to HIV care and treatment and retention on ART. For each indicator, each country’s policy was categorized into one of the following: (i) has explicit policy; (ii) has implicit policy or policy has caveats or exceptions; (iii) is unclear whether policy exists or policy conflicts with other policies; or (iv) does not have policy. We also assessed whether the policy was consistent with WHO guidance or a country standard that went beyond such guidance.

## Results

A total of 120 policy documents with guidance relevant to the indicators were identified and reviewed; references are available from the author.

### Access to testing

**Fig. 2:**
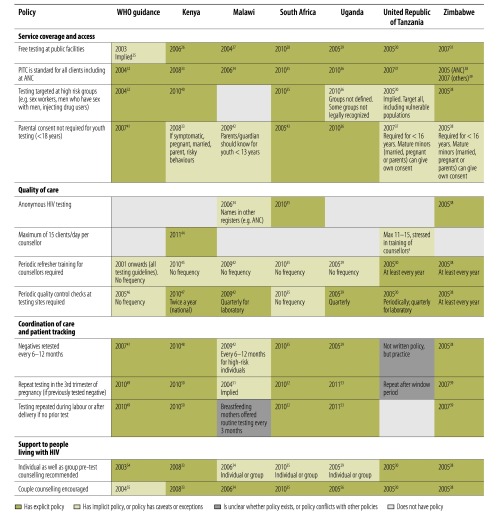
WHO guidance and policies in six African countries influencing access to HIV testing, 2003 to mid-2013 ^25^^–^^56^

ANC: antenatal care; HIV: human immunodeficiency virus; PITC: provider-initiated testing and counselling for HIV; WHO: World Health Organization.

^a^ Information from key informant interview.

Fig. 2 summarizes policies influencing access to HIV testing. Policies in the six countries were generally consistent and explicitly or partially adhered to the policy indicators, including provision of free testing services and provider-initiated testing and counselling. Malawi was the only country with no policy targeting testing among high-risk groups; while Uganda and the United Republic of Tanzania did not define the high-risk groups. Only South Africa and Uganda had explicit policies enabling minors to access testing without parental consent.

Indicators related to quality of care were more variable. Anonymous HIV testing was guaranteed only in South Africa and Zimbabwe, whereas in Kenya, Uganda and the United Republic of Tanzania names could be recorded in registers to facilitate patient management. Various WHO documents emphasized confidentiality and protection from discrimination, but did not offer explicit guidance on maintaining anonymity. Only Kenya and the United Republic of Tanzania had policies limiting the number of testing sessions that counsellors can perform per day. While all countries stipulated the need for periodic refresher training for counsellors and quality control checks at sites, they varied in stated frequency, with the United Republic of Tanzania and Zimbabwe most explicit about how often retraining is required.

Policies influencing coordination of care and patient tracking were more ambiguous in the United Republic of Tanzania where there was no clear policy on repeat testing intervals for negatives or on repeat testing during pregnancy, labour or after delivery. Malawi was also ambiguous in this area, with repeat testing for negatives advised every 6–12 months for high-risk individuals and no explicit policy on repeat testing during pregnancy.

Regarding patient support, Malawi, South Africa and Uganda also stipulated that pretest HIV counselling be conducted either individually or in groups; others recommended at least one individual session. While all countries promoted couple counselling, this was not explicit in WHO guidance.

### Access to care and treatment

**Fig. 3:**
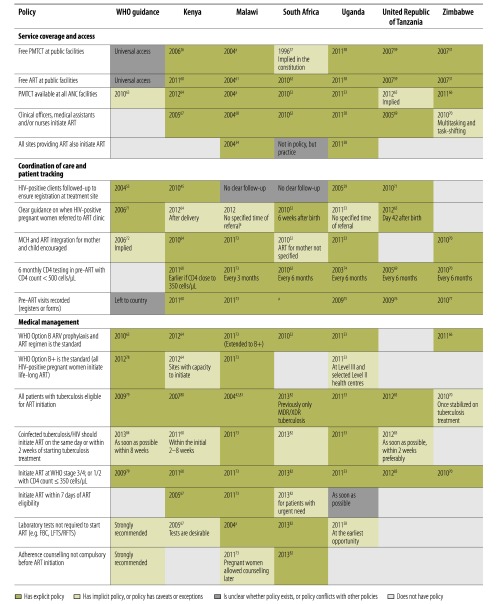
WHO guidance and policies in six African countries influencing access to HIV care and treatment, 2003 to mid-2013 ^26^^,^^29^^,^^31^^,^^34^^,^^42^^,^^45^^,^^52^^–^^55^^,^^57^^–^^84^

ANC: antenatal care; ART: antiretroviral therapy; ARV: antiretroviral; CD4: cluster of differentiation 4; FBC: full blood count; HIV: human immunodeficiency virus; LFTS/RFTS: liver function/renal function tests; MCH: maternal and child health; MDR/XDR: multi-drug resistant/extensively-drug resistant; PMTCT: prevention of mother-to-child transmission; WHO: World Health Organization.

^a^ Information from key informant interview.

Fig. 3 summarizes policies influencing access to care and treatment services, which varied more across countries. Free public sector access to PMTCT and ART was guaranteed everywhere, either explicitly in HIV policies or national health policies or implied in the national constitution, although WHO documents only implied free public sector access through promotion of universal access to HIV services. All countries promoted PMTCT availability within antenatal care and all allowed task-shifting of ART initiation to clinical officers, medical assistants or nurses (albeit with important variations in year of policy formulation, with Kenya, Malawi and the United Republic of Tanzania allowing task shifting as early as 2004/2005). Only Malawi and Uganda had explicit policies stating that all sites providing ART should also be able to initiate ART.

All countries had explicit policies on the need for CD4+ T-lymphocyte (CD4+ cells) testing at least every six months in the pre-ART phase and all recorded pre-ART visits in patient registers or forms. Only Kenya, Uganda and the United Republic of Tanzania had explicit policies on patient follow-up to ensure registration at treatment sites. All countries except Zimbabwe stipulated the need for PMTCT-ART referral, but only South Africa and the United Republic of Tanzania indicated when this referral should occur (six weeks after birth). Service integration between maternal and child health and ART was encouraged explicitly in Kenya, Malawi, Uganda and Zimbabwe.

Most countries adhered to WHO’s 2010 Option B regimen for PMTCT (provision of triple drug therapy to the mother during pregnancy until delivery or cessation of breastfeeding), except the United Republic of Tanzania which still adhered to WHO’s Option A regimen at the time of the review (single dose drug therapy with zidovudine [AZT] during pregnancy, triple therapy at onset of labour using nevirapine, AZT and lamivudine [3TC], followed by dual drug therapy for seven days postpartum with AZT and 3TC). Malawi was the only country to have explicitly adopted WHO’s Option B+ regimen (initiation of life-long triple ART therapy during pregnancy) for all women in 2011, which was earlier than WHO guidance. Roll-out of Option B+ in Kenya and Uganda was dependent on the capacity of the health facility to initiate triple therapy.

Most countries had explicit policies allowing all people co-infected with HIV and tuberculosis to initiate ART, but differed markedly in their year of uptake of this policy, ranging from 2004 in Malawi, to 2013 in South Africa. Country guidance varied on tuberculosis and HIV treatment initiation: Malawi and Uganda stated that co-infected patients must initiate ART on the same day or within two weeks of starting tuberculosis treatment (going beyond WHO guidance of within eight weeks); while Kenya and the United Republic of Tanzania stated that treatment should preferably be initiated within two weeks. While all countries shared the standard ART initiation criteria, only Kenya and Malawi stipulated explicitly that ART should be initiated within seven days of being found eligible for treatment. Malawi and South Africa also had more liberal policies allowing ART initiation with minimum requirements for tests and counselling. Malawi has not required laboratory tests for initiation since the beginning of the treatment programme (2004). South Africa brought in this change in 2013 and stated that adherence counselling should not be compulsory before initiation.

### Retention on ART

**Fig. 4:**
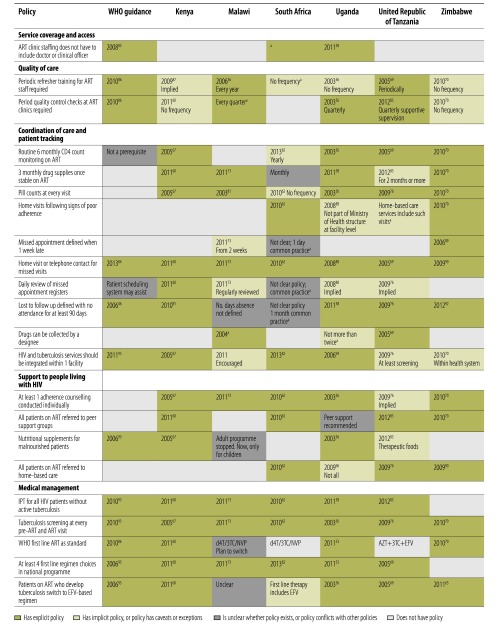
WHO guidance and policies in six African countries influencing retention on ART, 2003–mid-2013 ^36^^,^^46^^,^^53^^,^^56^^,^^58^^,^^60^^,^^62^^,^^67^^,^^69^^,^^70^^,^^73^^,^^76^^,^^81^^–^^96^

Fig. 4 summarizes policies influencing retention on ART. Stipulating that ART clinics include a doctor or clinical officer could be a barrier to access in resource-constrained contexts. This requirement is still applied in Kenya, Malawi, the United Republic of Tanzania and Zimbabwe (even though task-shifting for initiation occurred). Quality indicators also varied, with staff retraining and quality control intervals varying or not made explicit; for example no quality control was required after initial accreditation in South Africa, versus quarterly checks in Malawi, Uganda and the United Republic of Tanzania.

3TC: lamivudine; ART: antiretroviral therapy; AZT: zidovudine; CD4: cluster of differentiation 4; d4T: stavudine; EFV: efavirenz; HIV: human immunodeficiency virus; IPT: isoniazid preventive therapy; NVP: nevirapine; WHO: World Health Organization.

^a^ Information from key informant interview.

There were also important differences in coordination of care and patient tracking. Since the beginning of the treatment programme, Malawi did not promote routine CD4 monitoring, unlike all other countries where yearly or six-monthly monitoring was standard. While most countries advised that stable patients receive a three-month supply of drugs, two months of supplies were stipulated in the United Republic of Tanzania and one month in South Africa. All countries promoted regular pill counts, but only South Africa and Zimbabwe had explicit policies on home visits following signs of poor adherence – others recommended home visits or telephone contact following missed appointments. Only Kenya explicitly recommended daily register reviews to identify missed appointments. Definitions of missed appointments and loss to follow-up varied: Zimbabwe was most reactive with a missed appointment defined as a one-week delay, followed by Malawi where a two-week absence triggered action. Most countries defined loss to follow-up or defaulting as no attendance within 90 days of the last visit, but the number of days was not defined in Malawi and was unclear in South Africa. Malawi, Uganda and the United Republic of Tanzania also allowed drug collection by designees (treatment partners/guardians).

Support to those on ART varied. All countries explicitly or implicitly recommended individual adherence counselling, but Malawi and Uganda did not stipulate referral to peer-support. Kenya and Uganda provided nutritional support to ART patients; Malawi stopped this in adults due to lack of evidence. Only South Africa, the United Republic of Tanzania and Zimbabwe routinely referred all ART patients to home-based care programmes.

All sites recommended routine screening for tuberculosis, but Zimbabwe did not routinely provide isoniazid preventive therapy for tuberculosis prevention. All countries except Zimbabwe recommended at least four first-line ART regimen choices to patients, but not all complied with WHO’s 2010 recommended first line standard therapy (tenofovir, 3TC and nevirapine/efavirenz). Zimbabwe was the first to introduce WHO’s recommended regimen in 2010. All countries except Malawi had clear criteria for switching to efavirenz-based regimens for patients who develop tuberculosis.

## Discussion

Here we demonstrate wide variation in national HIV policies influencing service access and attrition through the diagnosis-to-treatment cascade across six countries with generalized HIV epidemics. Given that African countries usually adopt guidance from WHO, such a degree of policy variation is surprising.

Several indicators were consistent across all countries, but for many indicators, countries were progressive in selected areas and no country stood out as having a consistently enabling policy context that would have a decisive impact on service access and attrition. In Malawi, for example, policies designed to facilitate access to care (such as no requirements for routine CD4 testing, early adoption of the Option B+ PMTCT regimen, rapid ART initiation for those eligible), contrasted with certain policy gaps (such as targeted testing for high-risk groups, repeat testing during labour/after delivery, referral to peer support or home-based care for patients on ART). South Africa is another case where policies aiming to enhance access in some areas contrasted with policy gaps in others. South Africa had policies on anonymous HIV testing, home visits following signs of poor adherence and few barriers to starting ART (no required laboratory tests, adherence counselling, or physician presence in ART clinics), but lacked policies on quality control for ART, provision of three-monthly ART supplies, guidance on missed appointments or loss to follow-up and compliance with WHO first-line regimen standards. In the United Republic of Tanzania, an enabling policy environment for retention of patients in care and treatment contrasted with the slow adoption of WHO Option B+ and ART regimens containing tenofovir (both subsequently adopted in September 2013), as well as weaknesses in repeat testing intervals.

There were also important differences in the timing of policy implementation in some indicators. Malawi adopted Option B+ in 2011 (before WHO guidance) and has not required laboratory tests for ART initiation since 2004 (versus South Africa, which made this change in 2013). Policies to trace missed appointments with home visits or phone contacts vary in dates of implementation from 2004–2005 in Malawi and the United Republic of Tanzania, to 2011 in Kenya. While countries often lagged behind WHO in national policy adoption, in some instances countries went beyond WHO standards; notable examples included policies related to pre-ART-CD4 monitoring intervals, rapid initiation of ART, task-shifting for ART initiation, drug resupply intervals, pill count recommendations, drug collection by designees, referral to peer support and home-based care. The fact that WHO had no explicit guidance on such topics may contribute to the differences in adoption of policies across countries.

Policies are likely to differ in their potential impact on service access and attrition. We are unable to judge, therefore, how policy differences are likely to influence mortality. There seems to be little correlation between policy profile and service coverage (Table 1). Our analysis did not attempt to weight policies – as this is likely to be highly subjective. One might expect that policies on patient coordination and tracking (e.g. pre-ART monitoring, timely initiation of ART and adherence monitoring) have greater impact than policies on quality of care (e.g. staff training or quality audits). The relative emphasis on different policies also varied; some indicators were only mentioned once in one document, while others were core tenets of HIV service delivery and repeatedly mentioned.

We attempted to frame all policies as designed to increase service access and reduce attrition, but the direction of effect was not always clear-cut. For example, anonymous testing may promote access to diagnosis by reducing stigma, but may hamper linkage to care. Group pretest counselling may facilitate test access but have quality implications. Requiring people co-infected with HIV and tuberculosis to stabilize on tuberculosis treatment before initiating ART may be beneficial only for patients with very low CD4 counts.^97^ Allowing certain sites to provide ART refills may also be advantageous, allowing patients to access drugs nearer home. Fast-tracking patients into care (with rapid ART initiation and no requirements for laboratory tests or adherence counselling) may increase immediate uptake of services, but could undermine long-term adherence if patients are inadequately prepared for treatment. Not insisting on routine CD4 monitoring of ART patients in Malawi forms an important aspect of the public health approach to scaling up access, but may have negative consequences for timely identification of treatment failure.

Our review has some limitations. First, establishing the precise date of policy enactment was challenging. Publication dates represent formal enactment, but key informants reported instances in which policies came into effect earlier. Certain policies did not reappear in more recent documents, casting doubt on their validity. Furthermore, countries that recently produced HIV guidelines (South Africa: 2013 ART guidelines; Kenya: 2012 PMTCT guidelines) may appear to have more ‘advanced’ policies than those currently in the process of updating their guidelines, including Malawi and the United Republic of Tanzania. Second, the extent to which all relevant policy indicators were captured is uncertain. Our systematic approach to develop tools and indicators attempted to minimize this, but there may be other factors that were missed. We did not investigate the broader national policy environment (Fig. 1). Factors such as national politics, laws and the criminal justice system, programme financing mechanisms and donor coordination have been shown to have strong influences on health seeking-behaviour and service response.^98^^–^^101^ Country-specific policy analyses will be needed to analyse these national influences in detail.

Interpreting these findings with regard to potential programme impact is challenging. In part this stems from the wide variation demonstrated in our analysis, but perhaps more importantly because policies are only the first step in programme delivery. Their effectiveness depends on service-level implementation, as well community-level factors.^99^^,^^100^^,^ Further analysis will examine how these different policies are implemented in ALPHA’s network of health and demographic surveillance sites. This will allow us to assess whether policy translates into practice and whether practice exceeds stated policies, as often claimed by our key informants. International efforts to monitor policy implementation – such as WHO’s estimates on ART policy implementation^24^ – are increasing and we hope that this review and its tools can support other efforts to track national policies.
